# Liver injury and recovery following radiation therapy for hepatocellular carcinoma: insights from functional liver imaging

**DOI:** 10.20517/2394-5079.2024.27

**Published:** 2024-09-08

**Authors:** Peter Zaki, Kanokphorn Thonglert, Smith Apisarnthanarax, Clemens Grassberger, Stephen R. Bowen, Joseph Tsai, Jonathan G. Sham, Bing-Hao Chiang, Matthew J. Nyflot

**Affiliations:** 1 Department of Radiation Oncology, University of Washington, Seattle, WA 98195, USA.; 2 Department of Radiology, Division of Radiation Oncology, Chulalongkorn University, King Chulalongkorn Memorial Hospital, Thai Red C Society, Bangkok 10330, Thailand.; 3 Department of Radiology, University of Washington, Seattle, WA 98195, USA.; 4 Department of Surgery, University of Washington, Seattle, WA 98195, USA.

**Keywords:** HCC, child-pugh, SPECT, IMRT, SBRT, proton therapy, recovery, reirradiation

## Abstract

**Aim:**

A critical need for hepatocellular carcinoma (HCC) management is understanding how the liver recovers following radiotherapy (RT). We hypothesized that functional liver imaging with ^99m^Tc-sulfur colloid (SC) SPECT/CT provides additional information on liver injury and recovery after RT compared to conventional imaging.

**Methods:**

The liver function of patients with HCC was assessed using ^99m^Tc-SC SPECT/CT imaging before and after definitive RT. The anatomical liver volume (ALV) was segmented on CT imaging. Liver function was measured as the total liver function (TLF) encompassing 30% of maximum SC uptake. Changes in ALV and TLF were compared to clinical characteristics.

**Results:**

Of 31 patients with evaluable post-RT SC SPECT/CT scans (total of 32), 23 had pre-treatment Child-Pugh (CP)-A and 9 had CP-B/C scores. The median follow-up post-RT was 57 days. The median change in ALV was −1.7% with no significant difference between CP-A and CP-B/C patients (*P* = 0.26) or between short- (32-99 days) and long-term (271-1120 days) follow-up imaging groups (*P* = 0.28). The median change in TLF post-RT was −24% and was significantly different between short- and long-term groups (−39% *vs*. 2%, *P* = 0.001) and between CP-A and CP-B/C patients (−19% *vs*. −57%, *P* = 0.002). TLF significantly decreased following treatment at all radiation dose levels, with the decline correlating with the dose (*P* < 0.001).

**Conclusion:**

Functional imaging provides additional information regarding liver injury and recovery following RT that conventional imaging cannot reveal. Patients with CP-A liver status showed less decline following RT and most had liver function near or above pre-treatment levels.

## INTRODUCTION

Understanding how the liver recovers following radiation therapy (RT) for hepatocellular carcinoma (HCC) patients is critical. The majority of patients with HCC have cirrhosis, which places them at risk for potentially life-threatening complications from liver-directed treatment^[1-5]^. While liver regeneration after surgical resection is well described^[6-14]^, little is known about liver recovery following RT. Unlike hepatic resection, where part of the liver is removed and an increase in volume thereafter can be appreciated with conventional imaging (e.g., CT or MRI), changes in volume, if any, following RT are more difficult to measure. Therefore, functional imaging may be beneficial to assess liver regeneration following RT.

Comprehending liver recovery following RT is of vital importance due to the high sensitivity of the normal liver to RT which can lead to potentially life-threatening radiation-induced liver disease (RILD)^[15,16]^. This is especially relevant when considering courses of reirradiation. The results of this study would be important to multidisciplinary teams including hepatobiliary surgical oncologists, medical oncologists, and radiation oncologists. The findings can provide insight into potential treatment-related toxicity to help guide decision making regarding the optimal, personalized management of each patient with HCC.

Various functional imaging methods to measure liver function have been reported^[17-19]^. ^99m^Tc-sulphur colloid (SC) single photon emission tomography (SPECT/CT) is well-established as a method for assessing liver function^[19-26]^. Our research group has experience in using SC SPECT/CT to spatially and semi-quantitatively analyze liver function before and after RT^[19,20,25,26]^. SC is processed by the Kupffer cells of the reticuloendothelial system in the liver, and SC uptake can be quantified using SPECT/CT ^[19-22]^. The purpose of this study was to assess the utility of longitudinal SC SPECT/CT imaging in describing functional liver changes at different time intervals following RT for HCC. Our hypothesis was that utilizing SC SPECT/CT offers insights into the dynamics of liver injury and the subsequent recovery post-RT that complement conventional imaging. These dynamics may then inform liver function dose-response models, dose tolerances, and reirradiation guidelines.

## METHODS

A retrospective chart review was performed to identify a cohort of patients with HCC who were treated with definitive RT at our institution between 2013 and 2023. Patients were included if they received SC SPECT imaging before and after the RT course to assess liver function. Patients received 30-67.5 Gy in 5-15 fractions with either photon- or proton-based RT. Clinical liver status [cild-Pugh (CP) score] was obtained from the medical record. Anatomical and functional liver metrics were assessed on SPECT/CT before and after treatment. Pre-treatment SPECT/CT scans were typically conducted within 4 weeks of starting RT. As there was a natural separation, scans 32-99 days post-treatment were labeled as short-term and those 271-1120 days post-treatment were categorized as long-term.

The anatomic liver volume (ALV) was segmented on CT for attenuation correction imaging obtained at the time of SPECT/CT. Rigid image registrations were performed and metrics obtained using MIM 7.1.4 (MIM Software Inc, Cleveland, OH, USA). Radiation dose maps were converted to equivalent dose in 2 Gy fractions, presuming an EQD2_2Gy_(α/β of 3). Dose subvolumes were generated on CT for volumes receiving 1-10 Gy, 10-20 Gy, 20-30 Gy, and 30+ Gy. Liver function was measured with total liver function (TLF) as described previously^[20,26]^. Briefly, the TLF is the product of the mean liver-to-spleen uptake ratio and the ratio of the functional liver volume encompassing 30% of maximum uptake divided by the ALV. Conceptually, TLF takes into account both the density and volumetric extent of liver function in patients, similar to the future liver remnant^[20]^. Additional details on this calculation were previously published, and the 30% was previously determined to be the optimal TLF cutoff for overall survival prediction and CP classification ^[20,26]^. Changes in liver size and function were compared to clinical characteristics including CP score. This study was approved by the institutional IRB.

### Statistical analysis

ALV and TLF were assessed as functions of CP class before and after RT as well as time from initiation of RT. For post-RT SC SPECT/CTs, time from initiation of RT was noted and dichotomized into short- *vs*. long-term. Changes in ALV and TLF were assessed using non-parametric tests and analyzed for potential associations among different groups [e.g., CP class, short- *vs*. long-term, RT modality, and planning target volume (PTV)-to-liver volume ratio] using Wilcoxon rank-sum tests. Changes in ALV and TLF were analyzed for both the whole liver and radiation dose-level groups. Changes in ALV and TLF were compared among different dose levels using Kruskall-Walis test. Correlation between changes in ALV and TLF was assessed using Spearman rank correlation tests. The use of non-parametric tests was a conservative approach because there were too few patients to clearly determine if the distribution was normal. Furthermore, non-parametric tests are more robust to outliers, which is particularly important when the groups are small. To ensure robust results, the analyses were repeated with parametric statistics and the results were robust, showing similar differentiation between groups. Statistical calculations were performed using R v4.3.2 (R foundation for statistical computing, Vienna, Austria) with *P*-values based on two-sided hypothesis tests^[27]^. Graphs were made using Stata v17 (StataCorp, College Station, TX, USA).

## RESULTS

### Clinical and imaging data

There were a total of 31 patients with available treatment plans as well as pre- and post-RT SC SPECT/CT. There were 23 patients with short-term scans, 9 with long-term scans, and one with both short- and long-term scans. In total, 63 SPECT/CT images were analyzed. The median interval of short-term SC SPECT/CT was 54 days (range 32-99 days) following initiation of RT and the median interval of long-term SC SPECT/CT was 434 days (range 271-1120 days). Regarding baseline liver function status, 23 cases had CP-A5/6, 8 had CP-B7/8, and 1 had CP-C10 scores. Median follow-up post-RT was 57 days (range 32-1120 days). The median PTV was 106 cc (range 22-802 cc). Eighteen cases received proton therapy while 14 underwent photon-based (SBRT or IMRT) therapy.

### ALV change after RT

The median pre-treatment ALV was 1525 cc (range 810-2749 cc) with no significant difference in ALV between CP-A and B/C patients (1534 *vs*. 1279 cc, *P* = 0.4) or short- and long-term groups (1545 *vs*. 1442 cc, *P* = 0.5). When evaluating change following RT, the median change in ALV was −1.7% (range −29% to 23%) with no significant difference between CP-A and CP-B/C patients (−1.8% *vs*. 3.3%, respectively, *P* = 0.26) or between short- and long-term groups (−2% *vs*. −4%, *P* = 0.28). There was no correlation between the increase or decrease in ALV and the change in TLF (*P* = 0.19). Changes in ALV were not associated with RT modality (*P* = 0.5) or PTV-to-liver volume ratio (*P* = 0.6).

### TLF change after RT

**Figure 1.**
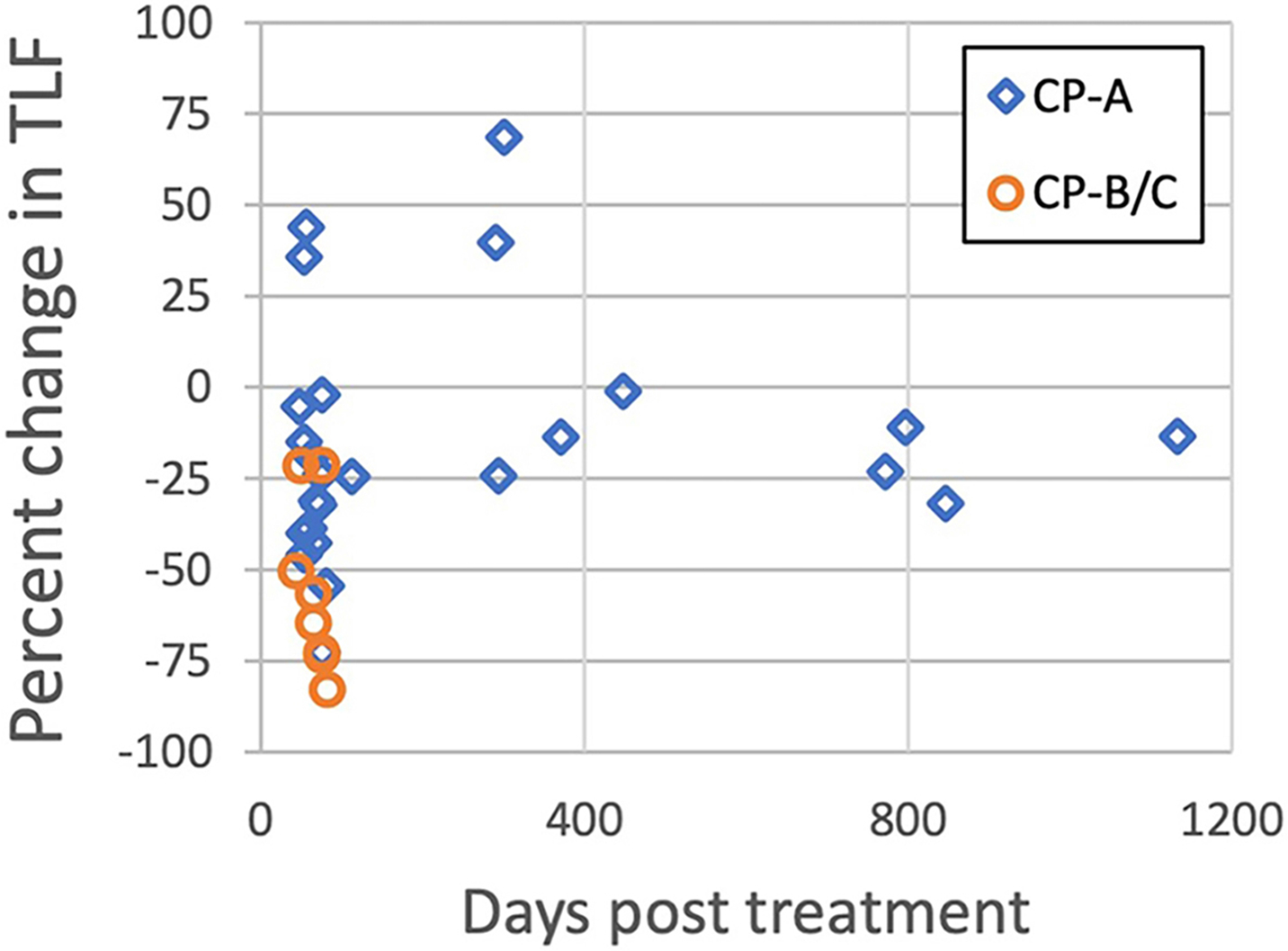
Change in liver function as measured by TLF as a function of time post-RT and CP status. CP-A patients showed less decrease in TLF than CP-B/C patients and a subset of CP-A patients showed a sizeable increase in TLF following RT. TLF: Total liver function; RT: radiotherapy; CP: child-pugh.

**Figure 2.**
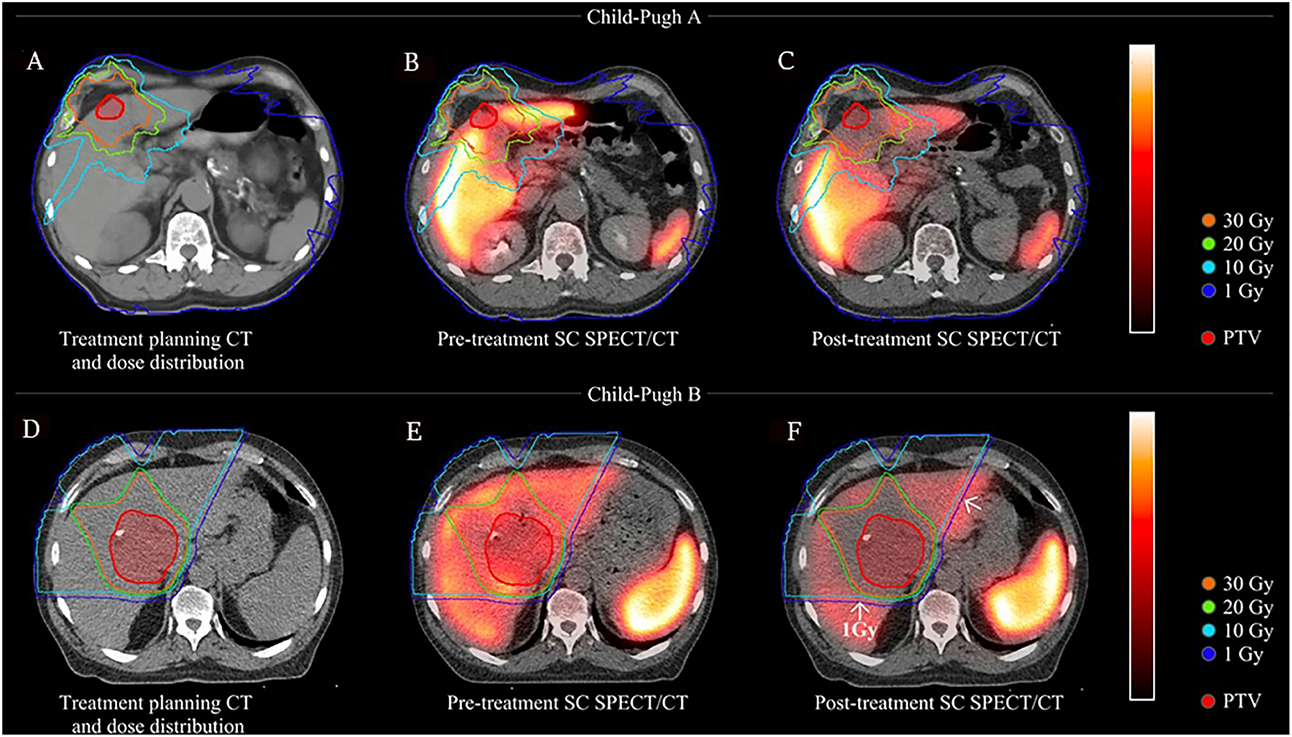
Visualization of spatial functional liver loss with SC SPECT/CT. 1st row (A-C): CP-A patient; 2nd row (D-F): CP-B patient; 1st column (A and D): radiation treatment plans with isodose lines; 2nd column (B and E): radiation treatment plans overlaid with pre-treatment SC SPECT/CT scans; 3rd column (C and F): radiation treatment plans overlaid with post-RT SC SPECT/CT scans demonstrating a higher threshold for function liver loss for the CP-A patient at 30 Gy and above (C) compared to the CP-B patient at 10 Gy and above (F). SC: Sulfur colloid; CP: child-pugh; RT: radiotherapy.

**Figure 3.**
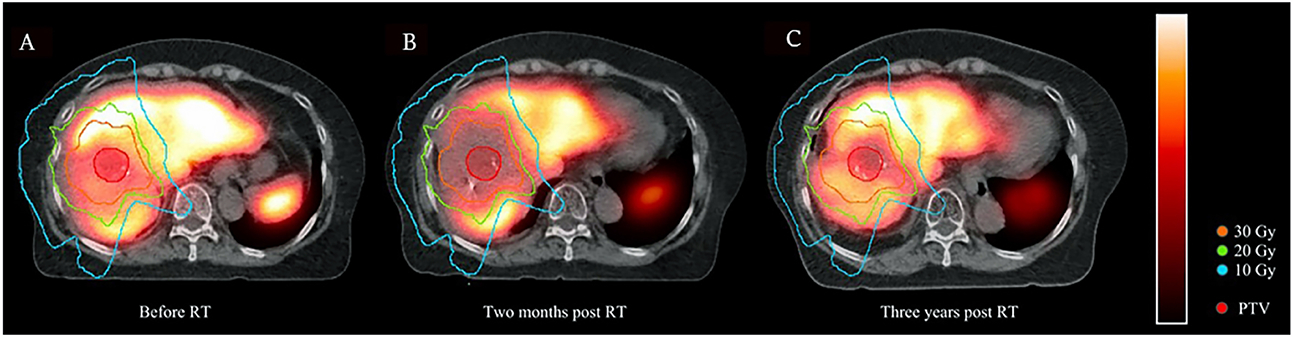
Functional liver recovery as imaged by serial longitudinal SC SPECT/CT in a CP-A patient who received multiple prior liver-directed therapies (chemoembolization, radiofrequency ablation, ethanol injection). (A) Radiation treatment isodose lines overlaid with pre-treatment SC SPECT/CT scan; (B) 2 months post-RT showing functional liver loss at 20 Gy and above; (C) 3 years post-RT showing functional liver recovery in regions that received 10-30 Gy. SC: Sulfur colloid; CP: child-pugh; RT: radiotherapy.

Pre-treatment median TLF was 0.79 (range 0.26-1.73) with no significant difference between short- and long-term groups (0.79 *vs*. 0.79, *P* = 0.3). TLF was significantly greater in CP-A compared to CP-B/C patients (0.93 *vs*. 0.58, *P* < 0.001). The median change in TLF was −24% (range −83% to 69%). The median TLF change was significantly different between short- and long-term groups (−39% *vs*. 2%, *P* = 0.001) and between CP-A and CP-B/C patients (−19% *vs*. −57%, *P* = 0.002) [Figures 1 and 2]. When analyzing only patients with CP-A status, median TLF change was still significantly different between the short- and long-term groups, (−28% *vs*. −9%, *P* = 0.02), suggesting an initial decline and the ability of well-compensated livers to functionally regenerate [Figure 3]. TLF change was not associated with RT modality (i.e., protons or photons, *P* = 0.26) or PTV-to-liver volume ratio (*P* = 0.06).

### Dose-level relationship to changes in ALV and TLF

Change in ALV did not significantly vary before and after RT or with radiation dose levels (*P* = 0.76). The median decline in ALV was −2.5%, −12%, −12%, and −6.9% for dose levels of 1-10 Gy, 10-20 Gy, 20-30 Gy, and > 30 Gy, respectively [Table 1]. ALV overall and within each sub-volume did not significantly differ between CP-A and CP-B/C groups. In terms of short- *vs*. long-term groups, ALV did not significantly vary overall but was significantly different for the 20-30 Gy and > 30 Gy dose levels.

TLF decreased significantly at all dose levels following treatment, and the median decline was greater at higher dose levels (−19%, −38%, −60%, and −76%, respectively, *P* < 0.001) [Table 2 and Figure 2]. When looking at dose-level groups, the change in TLF was significantly different between short- and long-term groups for overall TLF and dose > 20 Gy, but this dose-level specific difference was not significant when comparing only short- and long-term CP-A patients (data not shown). For all dose levels, patients with CP-B/C status had a greater decline in median TLF than patients with CP-A status.

**Figure 4.**
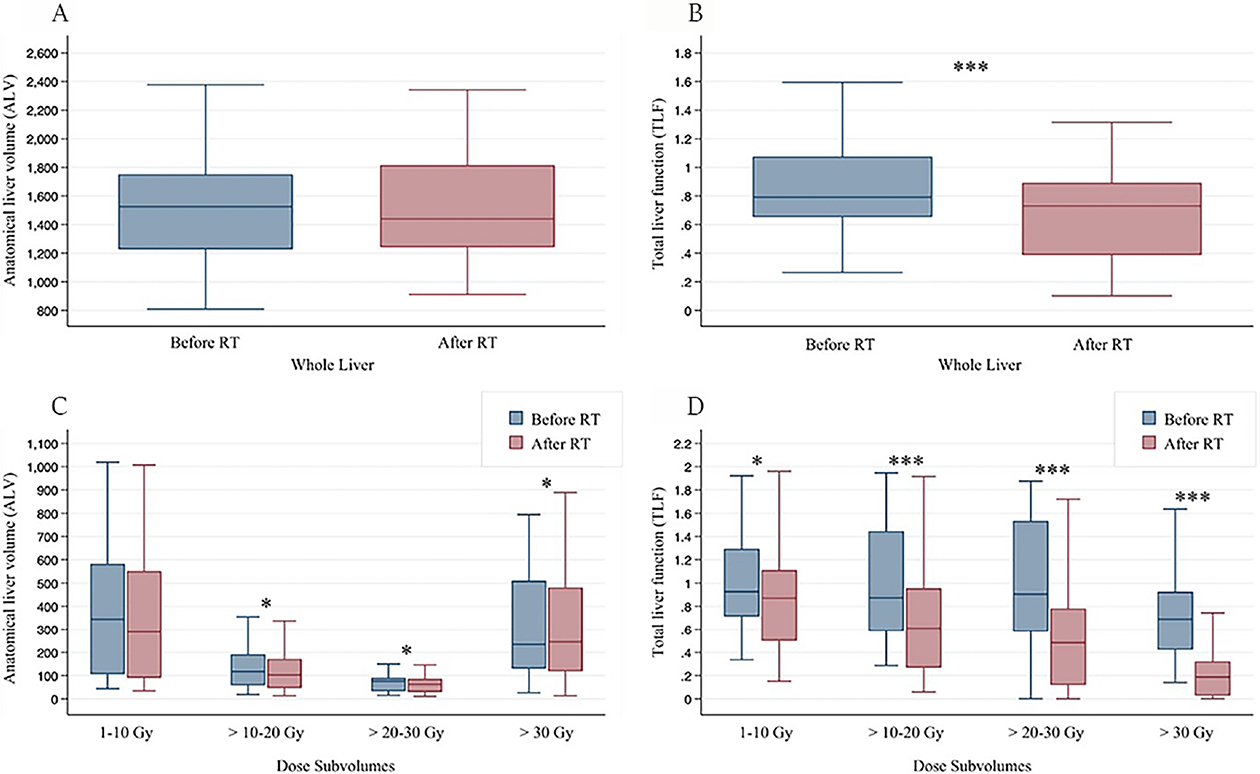
Changes in ALV and TLF after RT (A and B) and as a function of radiation dose levels (C and D). Median, interquartile range, and range are depicted. *P < 0.05; **P < 0.01; ***P < 0.001; ALV: anatomical liver volume; TLF:total liver function; RT: radiotherapy.

**Figure 5.**
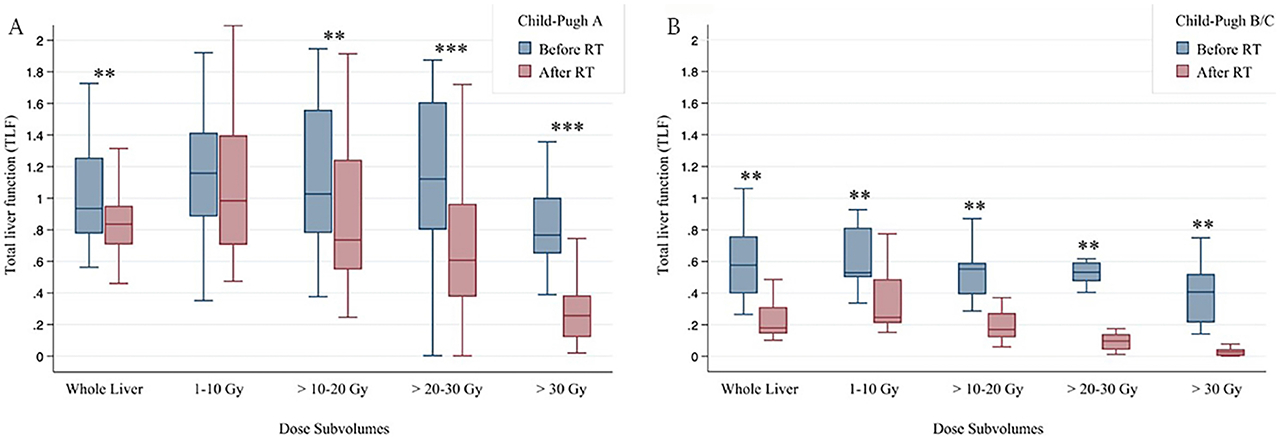
Changes in TLF as a function of radiation dose levels between CP-A patients (A) and CP-B/C patients (B). Median, interquartile range, and range are depicted. *P < 0.05; **P < 0.01; ***P < 0.001; TLF: total liver function; CP: child-pugh.

When comparing pre- and post-RT changes between ALV and TLF, no significant change was seen in ALV, whereas significant change was seen in TLF [Figure 4]. When analyzing individual dose sub-volumes, there were modest changes in ALV only at higher dose levels (> 10 Gy), whereas TLF showed decline even in the 1-10 Gy dose sub-volume. Figure 5 further stratifies change in TLF by CP status and shows that patients with CP-B/C scores had a more significant decline in TLF even at low dose levels of 1-10 Gy.

## DISCUSSION

Sulfur colloid SPECT/CT is a useful tool for liver functional imaging that is correlated to markers of liver function and can be used to guide the application of external beam RT. Our study found that functional liver imaging with SC SPECT/CT can yield additional insights into liver injury and recovery following RT that are not apparent on conventional imaging with CT.

Firstly, SC SPECT/CT was consistently able to detect spatial functional changes in the liver shortly (e.g., approximately one month) following RT, which cannot be appreciated by volume changes on radiographic analysis alone. Secondly, patients with well-compensated (CP-A status) liver function had less functional decline than CP-B/C patients following RT. CP-B/C patients had significant functional decline even in areas with low-dose (1-10 Gy) exposure. Thirdly, the regenerative capability of the liver was seen in areas receiving different radiation dose levels, with the most recovery in areas receiving the least radiation dose (< 10 Gy) and progressive impairment in regions receiving higher doses of radiation. Lastly, our limited data suggest that in some patients, there is the potential for long-term liver recovery at near or above pre-treatment function following RT. Together, these findings can help guide clinical decision making, particularly when personalizing a RT plan for HCC patients who may have cirrhosis and prior liver-directed treatments, including prior external beam RT.

In the current study, ALV as visualized on CT imaging was overall unchanged in the short- and long-term groups. The post-RT ALV was approximately only about 10% less than the pre-RT ALV for dose sub-volumes > 10 Gy, although more apparent in the long-term group [Table 1 and Figure 4]. This observation can potentially be explained by a gradual decrease in both tumor size and the volume of adjacent normal liver tissue, which may be negligible relative to the total liver volume or could be counterbalanced by the compensatory growth of non-irradiated liver tissue. Prior research supports this theory of compensatory liver growth following RT^[18,28,29]^. For example, Rim *et al*. assessed 77 patients with HCC who received RT. At a median follow-up of 50 days, patients with right hepatic lobe tumors showed no significant change in total liver volume; there was a decrease in tumor volume with an increase in non-tumor liver volume and future liver remnant hypertrophy^[28]^. However, not all patients experienced compensatory liver growth. Patients with left hepatic lobe tumors also showed no significant change in total liver volume and decrease in tumor volume, but conversely, no increase in nontumor liver volume or future liver remnant hypertrophy. The authors attributed this difference to the possibility that the right hepatic lobe is typically larger and may be sufficient to perform liver functions. Our study did not assess if ALV or TLF changes were related to the location of the liver tumor since the right or left liver lobes could not be reliably identified without intravenous contrast, which was not performed with SPECT/CT imaging. Similar to our study, the study by Rim *et al*. found a further decrease in total liver volume in the long term (median of approximately 400 days), although only statistically significant in subgroups of patients^[28]^. While some volumetric changes may be noted in these studies, there remain limited data from conventional imaging to formulate an understanding of the *functional* changes of the liver post-RT.

In contrast to the lack of significant change in total ALV, overall TLF significantly changed with RT. Post-RT TLF was significantly less than pre-RT TLF, ranging from about −20% at doses < 10 Gy to about −80% at doses > 30 Gy [Table 2 and Figure 4]. The median TLF change was significantly different between short- and long-term groups, suggesting more functional liver recovery in the long term following RT. The current study also found that baseline liver status affects functional loss following RT. Overall, most CP-A patients had less decline in TLF than CP-B patients and experienced more recovery of liver function near or above baseline [Figure 1]. For CP-B/C patients, even low RT doses (1-10 Gy) led to large reductions (approximately 50%) in liver function, in contrast to CP-A patients [Table 2 and Figure 5]. These findings provide insight into the functional liver changes following RT which would not have been appreciable with conventional imaging alone.

Prior studies have investigated the use of functional liver imaging in surgical patients. For example, Bennink *et al*. assessed ^99m^Tc-labeled mebrofenin hepatobiliary scintigraphy scans of 15 patients before, 1 day after, and 3 months after surgery^[17]^. Similar to our study, Bennink *et al*. found that functional imaging provides additional information regarding baseline liver function and regeneration than volumetric and laboratory analysis [indocyanine green (ICG) clearance] alone, particularly when liver function is inhomogeneous ^[17]^. In another study of 55 patients, De Graaf *et al*. further found that ^99m^Tc-labeled mebrofenin hepatobiliary scintigraphy scans could estimate the risk of postoperative liver failure^[30]^ Studies have shown that the timing for functional liver recovery after surgery appears to begin as early as 1 day postoperatively, with the majority of the recovery occurring within 2-3 months^[10,11,17,31]^. As previously mentioned, data exist describing the longitudinal nature of liver hypertrophy in non-irradiated liver as measured by size measurements on conventional imaging^[28]^. The timing of *functional* liver regeneration following RT, however, is less clear. Our study does not address this important topic, as we did not routinely perform multiple longitudinal SC SPECT/CT scans on individual patients, but it will be a focus of future studies in our group.

Following treatment, the liver may regenerate through various mechanisms. For surgical patients, the liver regeneration process, although not fully elucidated, is thought to include both hyperplasia and hypertrophy of mature hepatocytes and is mediated by both internal liver and extrinsic factors^[8,10-14]^. Various studies have found autocrine, endocrine, and paracrine signals, liver microenvironment (e.g., endothelial cells, intrahepatic lymphocytes, and Kupffer cells), and vascularity all to play a role in regeneration^[12,14,32]^. Limited data are available regarding mechanisms of liver regeneration after liver RT. Adachi *et al*.’s study delivered partial liver irradiation of 60 Gy to the anterior hepatic lobes with sparing of the posterior lobes. The study demonstrated progressive atrophy and significant fibrosis in the anterior lobes as detected by a standard laboratory test, while the posterior lobes exhibited hypertrophy without impaired liver function^[29]^. The study also noted a significant decrease in Ki-67 positive cells in the irradiated lobes early post-RT, with a significant increase in non-irradiated lobes, indicating compensatory liver growth through hyperplasia. In regards to the potential effects of baseline liver function on liver regeneration, Liu *et al*. harvested primary hepatocytes for repopulation experiments from normal rats and from rats with compensated or decompensated cirrhosis^[33]^. Interestingly, the study found that hepatocytes from rats with normal liver or compensated cirrhosis were immediately able to regenerate in a normal microenvironment while hepatocytes from rats with decompensated cirrhosis initially did not expand or show signs of function. However, after two months in the normal recipient liver, their function was re-established.

One limitation of our study is knowledge of the potential long-term liver recovery of patients with CP-B/C status following RT, as all the patients with long-term SC SPECT/CT in our study had CP-A liver status, which is understandable considering CP-A patients have a better prognosis^15^. It is unclear if the greater radiosensitivity of CP-B/C cirrhotic livers also translates to greater impairment of functional liver recovery after RT. Another limitation of this study is that clinical outcomes such as possible changes in clinical status, recurrence, and survival were not investigated. Endogenous markers related to liver function were not collected in this study. The sulfur colloid literature to date has focused primarily on correlations with histopathologic markers such as fibrosis, and correlation with new endogenous markers could elucidate the underlying mechanisms of liver damage and recovery following RT. However, TLF, akin to the concept of future liver remnant, has been correlated with albumin and bilirubin values, and associated with CP classification as well as overall survival prediction^[20,26]^. Although image registrations and structures were reviewed, anatomy can change with time following treatment and during respiration; thus, limitations of rigid registration may have affected TLF calculations within radiation dose sub-volumes. The application of deformable registration for longitudinal SC SPECT/CT" response would enable voxel-wise modeling of dose response and liver function and is an area of future investigation. Other limitations of our study include those intrinsic to retrospective design and relatively limited sample size. Further validation, including in prospective and external cohorts, is necessary.

There may be opportunities to exploit the findings of this study for RT planning. A correlation between liver recovery and CP status was observed, and the use of SC SPECT/CT" scans in our study provided spatial visualization of normal liver, which is particularly advantageous over standard laboratory tests when there is inhomogeneous liver function. A radiation oncologist may use this information to preferentially spare part of the liver when designing an RT plan or deciding on a preferred treatment modality. For instance, for well-compensated (CP-A) patients, a radiation oncologist may choose to use a technique such as volumetric modulated arc therapy which would not be able to avoid low-dose scatter in the surrounding liver, but for decompensated (CP-B/C) patients, one may prioritize minimizing liver exposure to even low-dose scatter by utilizing protons with specific beam angles, thereby mimicking the surgical concept of sparing the future liver remnant with capacity to hypertrophy^[34,35]^. In this study, 7 of the 8 CP-B/C cases received proton therapy whereas 11 of the 24 CP-A cases received protons. Logically, the decline in TLF for CP-B/C patients may have been more significant if protons were used less often. The current study showed that TLF change was associated with CP status but not associated with RT modality. In addition to the above findings, this study showed less liver recovery in regions with higher doses of radiation. Therefore, radiation oncologists are encouraged to reasonably minimize the volume of normal liver exposed to higher doses of radiation. While more research is needed, our study provides further insight into the timing of liver recovery after RT which would be relevant to clinicians when considering additional liver-directed therapies for patients.

In conclusion, functional imaging metrics reveal unique information about the potential functional reserve of irradiated livers compared to anatomic measurements. SC SPECT/CT is a sensitive marker of functional change following RT and reveals patterns of liver function that are not visible on anatomic CT at early time points. These data imply that functional liver imaging may more accurately assess the regenerative potential of irradiated and non-irradiated volumes of liver, which may be useful in scenarios when prediction of the remaining function of the liver after treatment becomes critical. Patients with better baseline liver status (CP-A) had less decline in liver function than CP-B/C patients overall and at all dose levels. In particular, CP-A patients had preserved liver function in low-dose regions (1-10 Gy), while even low doses caused a significant decline of function in CP-B/C patients. In a limited subset of patients with long-term imaging follow-up (9+ months), our data suggest that many well-compensated (CP-A) cirrhotic patients may recover nearly full liver function following RT. Further studies are needed to evaluate the dynamics and magnitude of liver recovery following RT, particularly in less compensated livers.

## Figures and Tables

**Table 1.** Change in ALV for the whole liver was similar between short- and long-term follow-ups, with only significantly different changes in the higher dose region. Change in ALV was similar between CP-A and CP-B/C patients for the whole liver and across all dose sub-volumes

	Whole liver	1-10 Gy	> 10-20 Gy	> 20-30 Gy	> 30 Gy
All patients	−1.7% (−29% to 23%)	−2.5% (−53% to 20%)	−12% (−42% to 66%)	−12% (−51% to 49%)	−6.9% (−54% to 39%)
Short-term (*n* = 23)	−2% (−29% to 23%)	−3% (−53% to 20%)	−5% (−33% to 66%)	−8% (−46% to 49%)	−2% (−28% to −39%)
Long-term (*n* = 9)	−4% (−21% to 18%)	−9% (−34% to 16%)	−18% (−42% to 9%)	−24% (−51% to −1%)	−31% (−54% to −1%)
*P*-value	0.28	0.93	0.12	0.01*	< 0.001**
CP-A (*n* = 23)	−1.8% (−29% to 18%)	−1.7% (−53% to 20%)	−12% (−42% to 66%)	−13% (−51% to 49%)	−7.1% (−54% to 39%)
CP-B/C (*n* = 9)	3.3% (−12% to 23%)	−3.3% (−35% to 9%)	−4.8% (−33% to 6.7%)	−11% (−46% to 19%)	−0.4% (−28% to 11%)
*P*-value	0.26	0.84	0.64	0.91	0.28

Median and range are provided. *P < 0.05; **P < 0.01; CP: child-pugh; ALV: anatomical liver volume.

**Table 2.** Change in TLF was significantly greater at long-term imaging over the whole liver with trends toward greater recovery in individual higher-dose sub-volumes

	Whole liver	1-10 Gy	> 10-20 Gy	> 20-30 Gy	> 30 Gy
All patients	−24% (−83% to 69%)	−19% (−76% to 151%)	−38% (−82% to 62%)	−60% (−96% to 46%)	−76% (−99% to 38%)
Short-term (*n* = 23)	−39% (−83% to 2%)	−24% (−76% to 61%)	−44% (−82% to 30%)	−64% (−96% to 16%)	−91% (−99% to −34%)
Long-term (*n* = 9)	2% (−36% to 69%)	15% (−31% to 151%)	−13% (−73% to 62%)	−20% (−80% to 46%)	−37% (−88% to 38%)
*P*-value	0.001**	0.06	0.05	0.004**	0.01*
CP-A (*n* = 23)	−19% (−58% to 69%)	−11% (−63% to 151%)	−26% (−73% to 62%)	−48% (−89% to 46%)	−62% (−98% to 38%)
CP-B/C (*n* = 9)	−57% (−83% to −21%)	−47% (−76% to 2%)	−69% (−82% to 14%)	−83% (−96% to −49%)	−95% (−99% to −70%)
*P*-value	0.002**	0.008**	0.002**	< 0.001***	0.003**

Note: all nine patients in the long-term cohort were well-compensated (CP-A). Change in TLF was significantly greater for CP-A status for the whole liver and all dose sub-volumes. Median and range are provided. *P < 0.05; **P < 0.01; ***P < 0.001; TLF: total liver function; CP: child-pugh.

## References

[1] Cheng JC, Wu JK, Huang CM, Radiation-induced liver disease after radiotherapy for hepatocellular carcinoma: clinical manifestation and dosimetric description. Radiother Oncol 2002;63:41–5. 12065102 10.1016/s0167-8140(02)00061-0

[2] Hsin IF, Hsu CY, Huang HC, Liver failure after transarterial chemoembolization for patients with hepatocellular carcinoma and ascites: incidence, risk factors, and prognostic prediction. J Clin Gastroenterol 2011;45:556–62. 21666547 10.1097/MCG.0b013e318210ff17

[3] Liang SX, Zhu XD, Xu ZY, Radiation-induced liver disease in three-dimensional conformal radiation therapy for primary liver carcinoma: the risk factors and hepatic radiation tolerance. Int J Radiat Oncol Biol Phys 2006;65:426–34. 16690430 10.1016/j.ijrobp.2005.12.031

[4] Salem R, Lewandowski RJ, Mulcahy MF, Radioembolization for hepatocellular carcinoma using Yttrium-90 microspheres: a comprehensive report of long-term outcomes. Gastroenterology 2010;138:52–64. 19766639 10.1053/j.gastro.2009.09.006

[5] Teh SH, Nagorney DM, Stevens SR, Risk factors for mortality after surgery in patients with cirrhosis. Gastroenterology 2007;132:1261–9. 17408652 10.1053/j.gastro.2007.01.040

[6] Higgins G, Anderson R. Experimental pathology of liver: restoration of liver in white rat following partial surgical removal. Arch Path 1931:202. Available from: https://www.scienceopen.com/document?vid=57858414-5eff-4c8d-a028-ccb3fc1c44a6.[Last accessed on 9 Sep 2024].

[7] Bucher NLR, Scott JF, Aub JC. Regeneration of the liver in parabiotic rats. Cancer Res 1951;11:457–65. 14839642

[8] Wenneker AS, Sussman N. Regeneration of liver tissue following partial hepatectomy in parabiotic rats. Proc Soc Exp Biol Med 1951;76:683–6. 14844311 10.3181/00379727-76-18594

[9] Moolten FL, Bucher NL. Regeneration of rat liver: transfer of humoral agent by cross circulation. Science 1967;158:272–4. 6053886 10.1126/science.158.3798.272

[10] Bucher NL. Liver regeneration: an overview. J Gastroenterol Hepatol 1991;6:615–24. 1782378 10.1111/j.1440-1746.1991.tb00921.x

[11] Fausto N, Campbell JS, Riehle KJ. Liver regeneration. Hepatology 2006;43:S45–53. 16447274 10.1002/hep.20969

[12] Fausto N, Campbell JS, Riehle KJ. Liver regeneration. J Hepatol 2012;57:692–4. 22613006 10.1016/j.jhep.2012.04.016

[13] Andersen KJ, Knudsen AR, Kannerup AS, The natural history of liver regeneration in rats: description of an animal model for liver regeneration studies. Int J Surg 2013; 11:903–8. 23899538 10.1016/j.ijsu.2013.07.009

[14] Hadjittofi C, Feretis M, Martin J, Harper S, Huguet E. Liver regeneration biology: Implications for liver tumour therapies. World J Clin Oncol 2021;12:1101–56. 35070734 10.5306/wjco.v12.i12.1101 PMC8716989

[15] Lasley FD, Mannina EM, Johnson CS, Treatment variables related to liver toxicity in patients with hepatocellular carcinoma, child-pugh class a and b enrolled in a phase 1-2 trial of stereotactic body radiation therapy. Pract Radiat Oncol 2015;5:e443–9. 25899219 10.1016/j.prro.2015.02.007

[16] Mizumoto M, Okumura T, Hashimoto T, Evaluation of liver function after proton beam therapy for hepatocellular carcinoma. Int J Radiat Oncol Biol Phys 2012;82:e529–35. 22284041 10.1016/j.ijrobp.2011.05.056

[17] Bennink RJ, Dinant S, Erdogan D, Preoperative assessment of postoperative remnant liver function using hepatobiliary scintigraphy. Nucl Med 2004;45:965–71. Available from: https://jnm.snmjournals.org/content/45/6/965.short.[Last accessed on 9 Sep 2024] 15181131

[18] Su TS, Li LQ, Liang SX, A prospective study of liver regeneration after radiotherapy based on a new (Su’s) target area delineaton. Front Oncol 2021;11:680303. 34513671 10.3389/fonc.2021.680303 PMC8426619

[19] Bowen SR, Saini J, Chapman TR, Differential hepatic avoidance radiation therapy: proof of concept in hepatocellular carcinoma patients. Radiother Oncol 2015;115:203–10. 25934165 10.1016/j.radonc.2015.04.011 PMC4587568

[20] Bowen SR, Chapman TR, Borgman J, Measuring total liver function on sulfur colloid SPECT/CT for improved risk stratification and outcome prediction of hepatocellular carcinoma patients. EJNMMI Res 2016;6:57. 27349530 10.1186/s13550-016-0212-9 PMC4923007

[21] Hoefs JC, Wang F, Kanel G. Functional measurement of nonfibrotic hepatic mass in cirrhotic patients. Am J Gastroenterol 1997;92:2054–8. 9362191

[22] Zuckerman E, Slobodin G, Sabo E, Yeshurun D, Naschitz JE, Groshar D. Quantitative liver-spleen scan using single photon emission computerized tomography (SPECT) for assessment of hepatic function in cirrhotic patients. J Hepatol 2003;39:326–32. 12927917 10.1016/s0168-8278(03)00296-4

[23] Everson GT, Shiffman ML, Hoefs JC, ; HALT-C trial group. quantitative liver function tests improve the prediction of clinical outcomes in chronic hepatitis C: results from the hepatitis C antiviral long-term treatment against cirrhosis trial. Hepatology 2012;55:1019–29. 22030902 10.1002/hep.24752 PMC3298578

[24] Groshar D, Slobodin G, Zuckerman E. Quantitation of liver and spleen uptake of ^99m^Tc-phytate colloid using SPECT: detection of liver cirrhosis. J Nucl Med 2002;43:312–17. 11884489

[25] Price RG, Apisarnthanarax S, Schaub SK, Regional radiation dose-response modeling of functional liver in hepatocellular carcinoma patients with longitudinal sulfur colloid SPECT/CT: a proof of concept. Int J Radiat Oncol Biol Phys 2018;102:1349–56. 29932945 10.1016/j.ijrobp.2018.06.017

[26] Schaub SK, Apisarnthanarax S, Price RG, Functional liver imaging and dosimetry to predict hepatotoxicity risk in cirrhotic patients with primary liver cancer. Int J Radiat Oncol Biol Phys 2018;102:1339–48. 30170100 10.1016/j.ijrobp.2018.08.029

[27] R Core Team. R: a language and environment for statistical computing. Available from: https://www.R-project.org/. [Last accessed on 9 Sep 2024].

[28] Rim CH, Park S, Woo JY, Seong J. Compensatory hypertrophy of the liver after external beam radiotherapy for primary liver cancer. Strahlenther Onkol 2018;194:1017–29. 30105451 10.1007/s00066-018-1342-y

[29] Adachi T, Yoshida Y, Shibuya K, Partial liver irradiation in rats induces the hypertrophy of nonirradiated liver lobes through hepatocyte proliferation†. J Radiat Res 2023;64:693–701. 37427542 10.1093/jrr/rrad051 PMC10354842

[30] de Graaf W, van Lienden KP, Dinant S, Assessment of future remnant liver function using hepatobiliary scintigraphy in patients undergoing major liver resection. J Gastrointest Surg 2010;14:369–78. 19937195 10.1007/s11605-009-1085-2 PMC2809979

[31] Gong WF, Zhong JH, Lu Z, Evaluation of liver regeneration and post-hepatectomy liver failure after hemihepatectomy in patients with hepatocellular carcinoma. Biosci Rep 2019;39:BSR20190088. 31383787 10.1042/BSR20190088 PMC6706596

[32] Richman RA, Claus TH, Pilkis SJ, Friedman DL. Hormonal stimulation of DNA synthesis in primary cultures of adult rat hepatocytes. Proc Natl Acad Sci 1976;73:3589–93. 1068471 10.1073/pnas.73.10.3589 PMC431163

[33] Liu L, Yannam GR, Nishikawa T, The microenvironment in hepatocyte regeneration and function in rats with advanced cirrhosis. Hepatology 2012;55:1529–39. 22109844 10.1002/hep.24815 PMC3700584

[34] Chuong MD, Kaiser A, Khan F, Consensus report from the miami liver proton therapy conference. Front Oncol 2019;9:457. 31214502 10.3389/fonc.2019.00457 PMC6557299

[35] Zaki P, Chuong MD, Schaub SK, Lo SS, Ibrahim M, Apisarnthanarax S. Proton beam therapy and photon-based magnetic resonance image-guided radiation therapy: the next frontiers of radiation therapy for hepatocellular carcinoma. Technol Cancer Res Treat 2023;22:15330338231206335. 37908130 10.1177/15330338231206335 PMC10621304

